# chromoWIZ: a web tool to query and visualize chromosome-anchored genes from cereal and model genomes

**DOI:** 10.1186/s12870-014-0348-6

**Published:** 2014-12-10

**Authors:** Thomas Nussbaumer, Karl G Kugler, Wolfgang Schweiger, Kai C Bader, Heidrun Gundlach, Manuel Spannagl, Naser Poursarebani, Matthias Pfeifer, Klaus FX Mayer

**Affiliations:** Plant Genome and System Biology (PGSB), Helmholtz Center Munich, D-85764 Neuherberg, Germany; Institute for Biotechnology in Plant Production, IFA-Tulln, University of Natural Resources and Life Sciences, A-3430 Tulln, Austria; Leibniz Institute of Plant Genetics and Crop Plant Research (IPK), OT Gatersleben, Corrensstraße 3, D-06466 Stadt Seeland, Germany

**Keywords:** Cereals, Bread wheat, Barley, *Brachypodium*, Rice, Comparative genomics

## Abstract

**Background:**

Over the last years reference genome sequences of several economically and scientifically important cereals and model plants became available. Despite the agricultural significance of these crops only a small number of tools exist that allow users to inspect and visualize the genomic position of genes of interest in an interactive manner.

**Description:**

We present *chromoWIZ*, a web tool that allows visualizing the genomic positions of relevant genes and comparing these data between different plant genomes. Genes can be queried using gene identifiers, functional annotations, or sequence homology in four grass species (*Triticum aestivum, Hordeum vulgare, Brachypodium distachyon*, *Oryza sativa*). The distribution of the anchored genes is visualized along the chromosomes by using heat maps. Custom gene expression measurements, differential expression information, and gene-to-group mappings can be uploaded and can be used for further filtering.

**Conclusions:**

This tool is mainly designed for breeders and plant researchers, who are interested in the location and the distribution of candidate genes as well as in the syntenic relationships between different grass species. *chromoWIZ* is freely available and online accessible at http://mips.helmholtz-muenchen.de/plant/chromoWIZ/index.jsp.

**Electronic supplementary material:**

The online version of this article (doi:10.1186/s12870-014-0348-6) contains supplementary material, which is available to authorized users.

## Background

Since the release of the sequenced genome of *Arabidopsis thaliana* in 2000 [1], more than 50 plant reference sequences have become available [2]. While the average genome size in Angiosperms is about 6 Gb [3], sequencing efforts have focused mainly on smaller-sized genomes (< 1 Gb), which serve as models for large and still unsequenced species or on more accessible crop plant genomes such as rice (*Oryza sativa*). The cereal species of the Pooideae subfamily, including bread wheat (*Triticum aestivum*), barley (*Hordeum vulgare*), and rice are among the most important crops and share a high degree of syntenic conservation on a genome-wide level [4,5]. Among the crops, hexaploid bread wheat (*T. aestivum*, 2n = 6x = 42, AABBDD) contains the largest and most complex genome with a size of roughly 17 Gb [6]. Despite its high economic relevance – 20% of the calories consumed by the world’s population derive from bread wheat – its genome has so far not been completely assembled. It has taken several years to provide a reference sequence for even one chromosome (3B, [7]), which by itself exceeds the genome size of rice almost 3-fold. Recently, shotgun sequencing and flow cytometry provided the basis for a gene annotation of the complete bread wheat genome comprising ~124 k gene models [6]. Furthermore, for selected chromosomes or chromosome arms, a physical map has been established and genetically anchored (e.g. 1A [8,9], 1BS [10], 3B [7,11], 6A [12]). For barley an anchored physical map that covers 3.9 Gb cumulative map length has been released [13,14], including 26 k high-confidence genes and comprises shotgun assemblies from three cultivars. Most shotgun contigs have already been anchored by population genetics. This approach, called POPSEQ [15], was also used to improve the anchoring of the physical map [13]. Like bread wheat and barley, *Brachypodium* (*Brachypodium distachyon*) also belongs to the Pooideae subfamily within the Poaceae family. It has a relatively small genome (~300 Mb) and has been widely used as a model organism to study the structure and evolution of other grass species [16]. Rice is another important member of the Poaceae family and represents one of the most important staple foods worldwide. To successfully integrate all the different resources, e.g. genetic information and gene expression measurements, for these crop species, heterogeneous datasets need to be combined. Therefore, tools and standards for interlinking anchored datasets are required (reviewed in [17]). One of the approaches for combining heterogeneous datasets is the “GenomeZipper” [4]. It establishes a virtual order of genes in plants without assembled chromosomes by exploiting the highly conserved synteny to smaller, already sequenced genomes. Large-sized syntenic regions, together with genetic marker sets enable an anchoring of most genes for larger-sized cereals including e.g. barley [14], rye (*Secale cereale*) [18] and *Aegilops tauschii* [19]. Since after the split from their common ancestor, the position of most genes was conserved, this approach provides robust approximations of the gene positions and order [20].

A small number of tools exist that allow users to inspect the genomic position of query genes in target genomes. For barley it is possible to map query sequences by using IPK Viroblast (http://webblast.ipk-gatersleben.de/) or barleymap (http://floresta.eead.csic.es/barleymap/). However, to our knowledge, no web-based tool exists that covers several genomes and allows calculating and visualizing the gene density along the chromosome. This is especially of importance when several dozen genes need to be mapped, e.g. for analyzing a quantitative trait locus (QTL). Transcriptome-oriented studies might reveal a set of gene candidates and the corresponding genomic position supports in removing false-positives gene candidates and defining the genetic or physical location of the QTL. None of the listed tools provide queries based on functional annotation or the integration of expression data. As part of the GenomeZipper, we have previously implemented a module ‘*chromoWIZ’* which was introduced to ease detection of syntenic regions for a yet unassembled genome and several sequenced and assembled genomes including *Brachypodium* [16], rice [21] and sorghum (*Sorghum bicolor*) [22]. Here, we describe the web-based version of *chromoWIZ* along with new features. Originally, *chromoWIZ* was restricted to local use as part of the GenomeZipper package and allowed a mapping of genes or shotgun contigs of one chromosome or chromosome arm against the reference genomes *Brachypodium*, rice and sorghum. To find genomic positions for genes of interest, in the latest, web-based version functional annotations and sequence homology can be used to find the corresponding regions within the corresponding genome. For grouped or clustered genes *chromoWIZ* now visualizes the physical position in a group-wise manner. In its latest version, *chromoWIZ* integrates the anchoring results of both the International Barley Genome Sequence Consortium (IBSC [14]), and the International Wheat Genome Sequencing Consortium (IWGSC [6]) and allows comparing sequences against the genomes of *Brachypodium* and rice. This tool is mainly designed for breeders and plant researchers, who are interested in the location and the distribution of candidate genes as well as in the syntenic relationships between different grass species. In order to illustrate the features of *chromoWIZ* and to explain the basic work-flows, we present different use cases. The application website can be accessed at: http://mips.helmholtz-muenchen.de/plant/chromoWIZ/index.jsp without any restrictions.

## Construction and content

*chromoWIZ* runs on a webserver at the PGSB site [23]. The tool’s back-end is implemented in the programming language Python. The front-end uses native HTML and Javascript for data visualization and navigation. Mapping information and gene information were collected from the official releases of the *Brachypodium*, rice, barley and bread wheat genomes [6,14,16,21]. For *Brachypodium* protein and coding sequences, as well as functional annotation information were collected from the PGSB database [23] using gene models’ version 1.2. For barley we integrated the datasets that were provided with the genetically anchored physical map [14], which is hosted at ftp://ftpmips.helmholtz-muenchen.de/plants/barley/public_data. For bread wheat, gene models from version 2.2 (ftp://ftpmips.helmholtz-muenchen.de/plants/wheat/IWGSC) were included. The MSU7 annotation has been integrated for rice [21]. More details for the currently used datasets and the corresponding updates are provided on the *chromoWIZ* web site.

## Utility

### Application of chromoWIZ

*chromoWIZ* allows visualizing the location of anchored genes along chromosomes on the basis of functional gene annotations, sequence homology or gene lists. So far, the web tool includes the crop species bread wheat (*T. aestivum*), barley (*H. vulgare*) and the closely related but much smaller *Brachypodium* (*B. distachyon*) and rice (*O. sativa*) genomes. Anchored genes are clustered together along the chromosome in non-overlapping genomic or genetic intervals, referred to as bins. In *Brachypodium* and rice, every bin represents one megabase (Mb) of non-overlapping chromosomal sequence. For barley 10 Mb and for bread wheat 5 CentiMorgan (cM) intervals are used. Bins are visualized as heat maps to enable an intuitive view along the entire chromosomes. The genomic positions in barley are highlighted relative to the anchored physical BAC contigs which were strung together to form virtual chromosomes. All genes within *chromoWIZ* are linked to external databases providing additional information on the gene models (e.g. for bread wheat EnsemblePlants http://plants.ensembl.org/Triticum_aestivum/Info/Index). The sequences of tagged genes within a bin can be downloaded in the FASTA format. To obtain the genomic location for genes of interest, referred to as “tagged genes”, *chromoWIZ* provides several search methods (Table 1): By sequence homology a set of query sequences can be mapped against the annotated gene models using nucleotide or protein BLAST searches, requiring a predefined e-value and sequence identity. Alternatively, if known, a list of species-specific gene identifiers can be directly provided instead of sequences. To query families of genes (e.g. genes sharing a specific Gene Ontology (GO) term or PFAM domain [24,25], an annotation-based approach has been included. The distribution of query genes is visualized by heat maps, which depict the relative distribution of the query-matching genes compared to the overall number of genes along the chromosomes. In addition, the overall gene distribution is shown, as the number of anchored genes varies between the different bins. To see whether certain chromosome (−arms) are enriched for tagged genes an enrichment analysis is provided. The significance of over-representation of genes tagged is assessed by a one-sided Fisher’s exact test and a Bonferroni adjustment of P values. Furthermore, labeled groups of genes can be included, e.g. genes being clustered or co-expressed or that were grouped together based on sequence similarity to allow for a group-wise visualization and analysis. The Data Manager is a part of *chromoWIZ* that enables the upload of various user-specific datasets and performs a validation of input data prior to integration into the *chromoWIZ* search interface. These data are subsequently only visible for the respective user and available for 24 hours before they are being automatically removed from the servers. Gene expression is an important factor for judging the relevance of candidate genes. In *chromoWIZ*, by using the Data Manager, users can optionally upload expression values for their genes of interest. Similar to expression data, information about differential expression can be provided. With expression data at hand, functional information can be combined with the genomic positions.

**Table 1:** **A variety of search features are provided by** ***chromoWIZ***

**Search feature**	**Description**	**Data needed**
Sequence similarity	Genes can be searched using homology either on nucleotide sequence level (BLASTN) or protein sequence level (BLASTP).	-
Gene identifier	List of gene identifiers as provided within the genome release.	-
Gene Ontology (GO) annotation	Genes can be searched based on their GO annotation.	-
PFAM annotation	Genes can be searched based on their PFAM annotation.	-
Expression variation	Gene expression levels need to vary across conditions in order to filter for interesting genes as quantified by using the coefficient of variation (sample standard deviation divided by the sample mean).	Expression matrix
Presence of expression	The expression has to surpass a custom expression threshold in at least one condition.	Expression matrix
Differential expression	Genes have to be in a list of genes being differentially expressed, as provided by the user.	List of differentially expressed genes
Gene clustering	Genes have to be in a certain group of clustered genes. Clustering information is provided by the user.	Gene to cluster linkage list

While some features are always available for all genomes, for the expression-based searches the user has to upload the corresponding data first.

The following use cases illustrate different aspects of *chromoWIZ*. The first use case describes how candidate genes can be mapped against the reference genome sequences using the gene identifiers, sequence-based searches or functional annotations. The second use case illustrates how a list of genes can be filtered based on their expression or by including information about differential expression. In the third use case we show how *chromoWIZ* allows highlighting syntenic regions between bread wheat and *Brachypodium* or barley. In the fourth use case we use published expression data to illustrate how the gene-to-group information can help in refining the genomic position of a resistance QTL. This is granted by transferring data from ancient to recent reference sequences. The fifth use case finally gives an example of how *chromoWIZ* can be applied for comparative genomic analysis.

### Use case 1: finding genes using identifiers, sequence similarity or annotations

**Figure 1:**
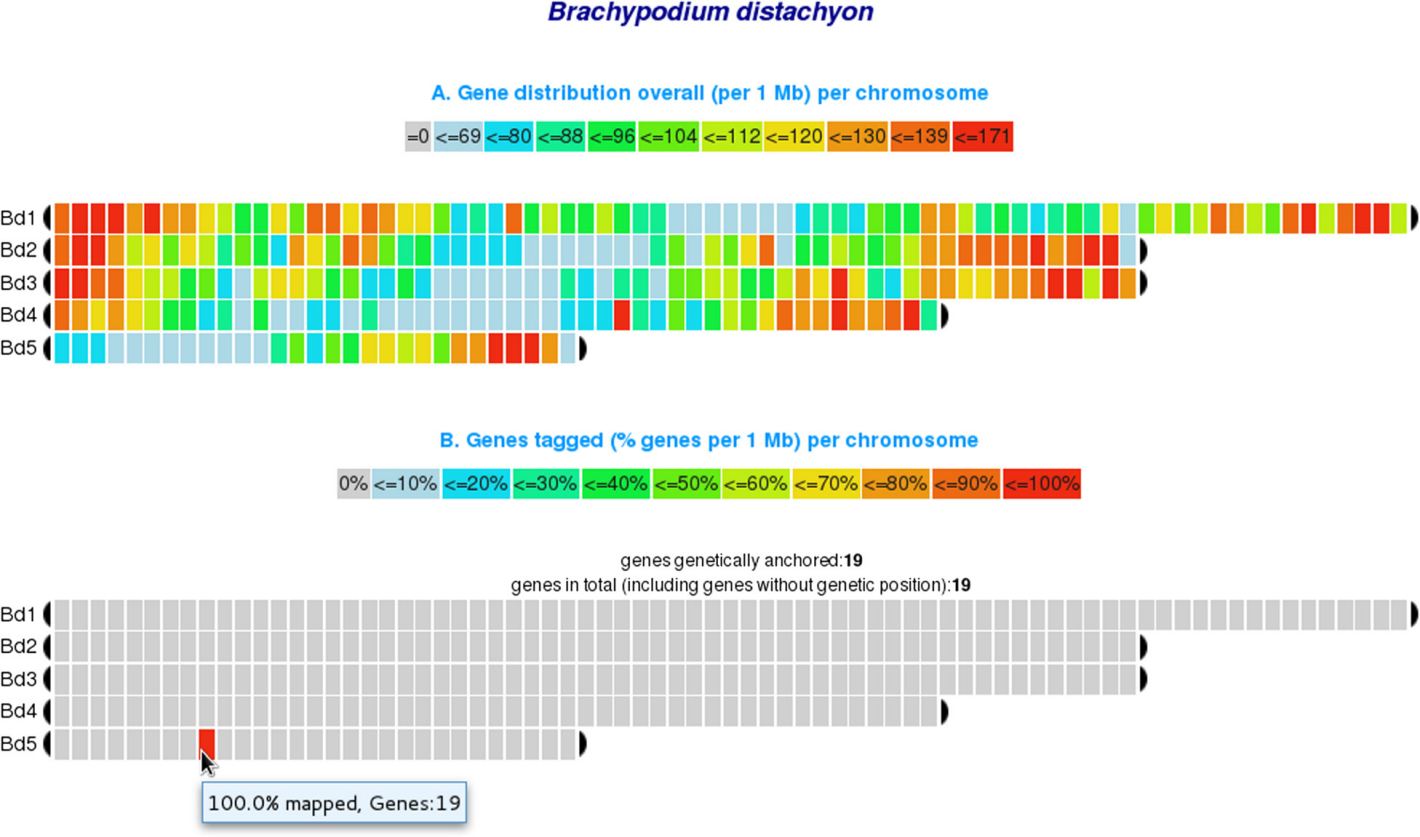
**Heat map visualization of gene density.** *chromoWIZ* visualizes the gene distribution of **(A)** all genes anchored as compared to **(B)** the number of genes matching the query criteria. The tooltip reports the relative and absolute number of tagged genes per bin.

One of the very basic functionalities of *chromoWIZ* is searching and visualizing genes by their identifiers. Given a set of species-specific gene identifiers their genomic position can be highlighted. In case no identifiers are available, an alternative approach is to provide sequence information for the corresponding genes. To illustrate this feature, we use the following example: A list of 19 gene identifiers from *Brachypodium*, preselected from a particular genomic region, was provided to the search interface (the gene identifiers are given in Additional file 1). *chromoWIZ* provides two outputs: First a heat map which depicts the number of all anchored genes along the chromosomes per bin (Figure 1A), and secondly, a heat map showing only the anchored genes that meet the query criteria (tagged genes) is shown (Figure 1B). For the given example the corresponding bin (bin9, 9-10 Mb) on chromosome 5 is highlighted. To illustrate the sequence-based search, we first extracted the gene sequences from this bin, by using the FASTA export functionality of *chromoWIZ*. This set of sequences was then provided to the search interface in order to perform a homology-based search. By only visualizing matches below an e-value of 10E-5, sequence identity of 100% and by requiring a best bidirectional match (flag ‘BBH’ has to be set) we again retrieved the bin containing the genes.

Besides the gene identifier and homology-based search, *chromoWIZ* also offers a search by gene annotation functionalities. A user might be interested in a particular gene family and would like to analyze whether members of that family have increased or decreased copy numbers compared to other genomes. One way to analyze differences in copy numbers is to compare the amount of genes on the basis of protein families (PFAM [25]) or Gene Ontology (GO [24]) terms and *chromoWIZ* includes annotation information from these sources. In the given example, we aimed at visualizing all genes that are annotated under the Gene Ontology (GO) term GO:0043565 (sequence-specific DNA binding) that e.g. comprises transcription factors. In *Brachypodium*, we found matches to 349 genes, in bread wheat matches to 421 (732 including genetically unanchored) genes, and in barley we found matches to 225 (340) genes.

### Use case 2: filtering for differentially expressed genes and usage of expression constraints

**Figure 2:**
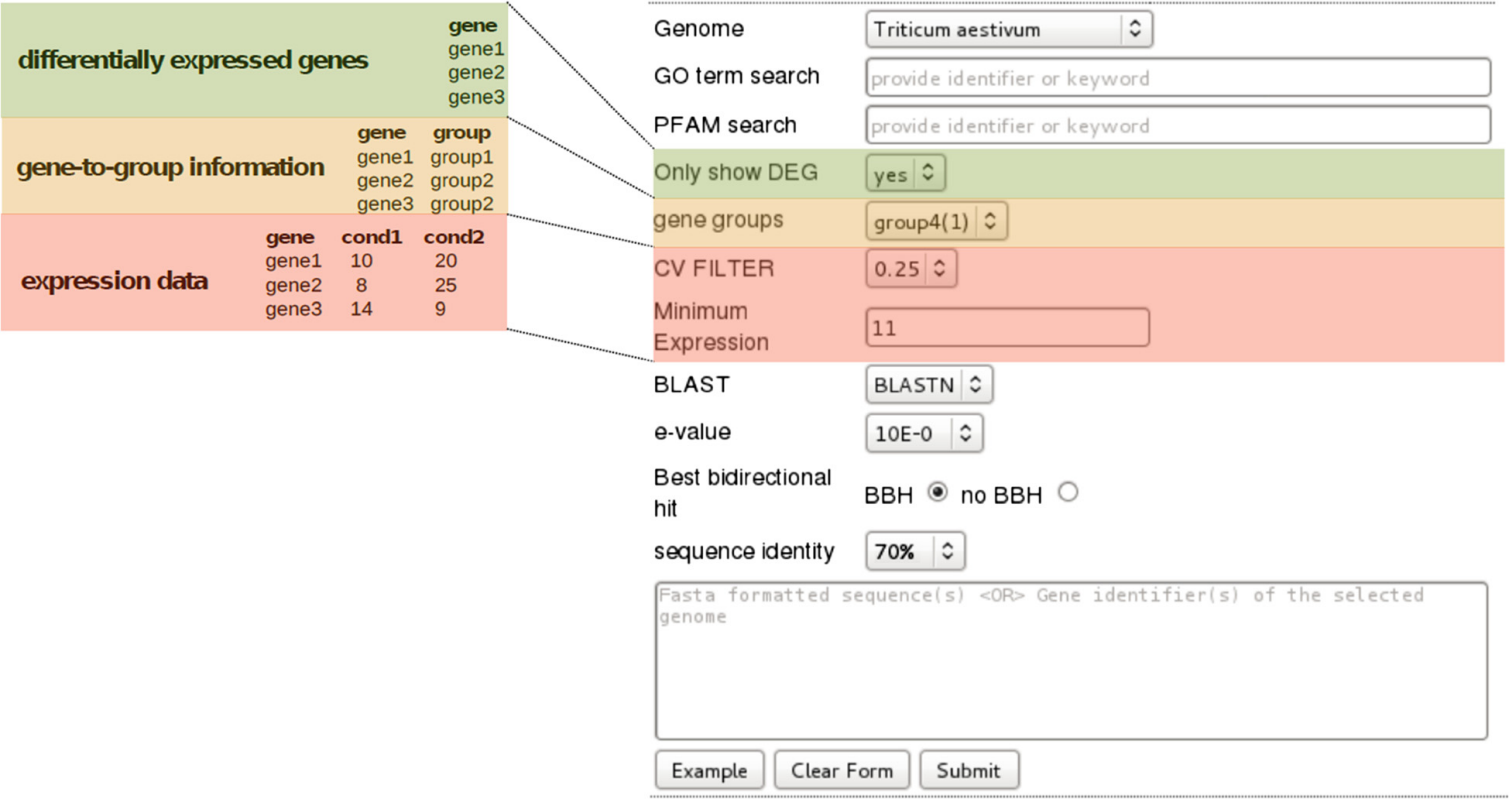
**Integration of gene expression information.** Gene expression information, lists of differentially genes, and/or gene-to-group mapping data can be uploaded for enabling expression-based querying of genes. The different color codes highlight the search options, which become available after uploading the corresponding data.

RNA-seq data is commonly used to analyze gene expression on a genome-wide level. It can efficiently be processed by means of analysis pipelines such as Cufflinks [26] or HTSeq [27]. After finding gene candidates based on their expression patterns it is often of interest to explore their respective genomic position. *chromoWIZ* provides features for combining expression data with positional information: **(i)** gene-to-group information can be provided. **(ii)** lists of differentially expressed genes can be included, and **(iii)** expression data of all genes can be integrated. Figure 2 shows the Data Manager input and the extended query features on the entry site, which are available once the data sets are included. Gene-to-group information is provided by an input file where the first column contains the gene identifier and the second column defines the group. The differentially expressed genes (DEGs) are provided via an input file that contains the gene identifiers. Also expression information can be provided in a file, where columns represent the conditions of interest. Details about the file formats are given on the *chromoWIZ* help page. When information about differentially expressed genes is included, the user can specify whether only differentially expressed genes should be queried. If expression information is included, genes can be filtered by two criteria: Either by a ‘Minimum expression’ criterion, meaning that at least in one condition the expression must exceed a given threshold. Alternatively, to find genes with expression variation across conditions, a user can set a ‘*CV’* (coefficient of variation, given by dividing the sample standard deviation by the sample mean) filter, to only keep genes with a minimum required *CV* value.

For illustration we extracted 692 barley transcripts that are differentially expressed between two Tibetan wild barley genotypes in response to low potassium treatment [28]. The transcript sequences as given in Table S3 of [28] (http://www.plosone.org/article/fetchSingleRepresentation.action?uri=info:doi/10.1371/journal.pone.0100567.s009) were mapped against the genetically anchored barley gene models using BLASTN (sequence identity greater than 95%, e-value of 10E-10, BBH criterion)*.* The 450 matching genes were compiled into a list of differentially expressed genes (Additional file 2) and uploaded by using the Data Manager. When searching barley for anchored differentially expressed genes we obtained 286 hits scattered across the different chromosomes.

### Use case 3: pronounced syntenic regions shared in grass species

**Figure 3:**
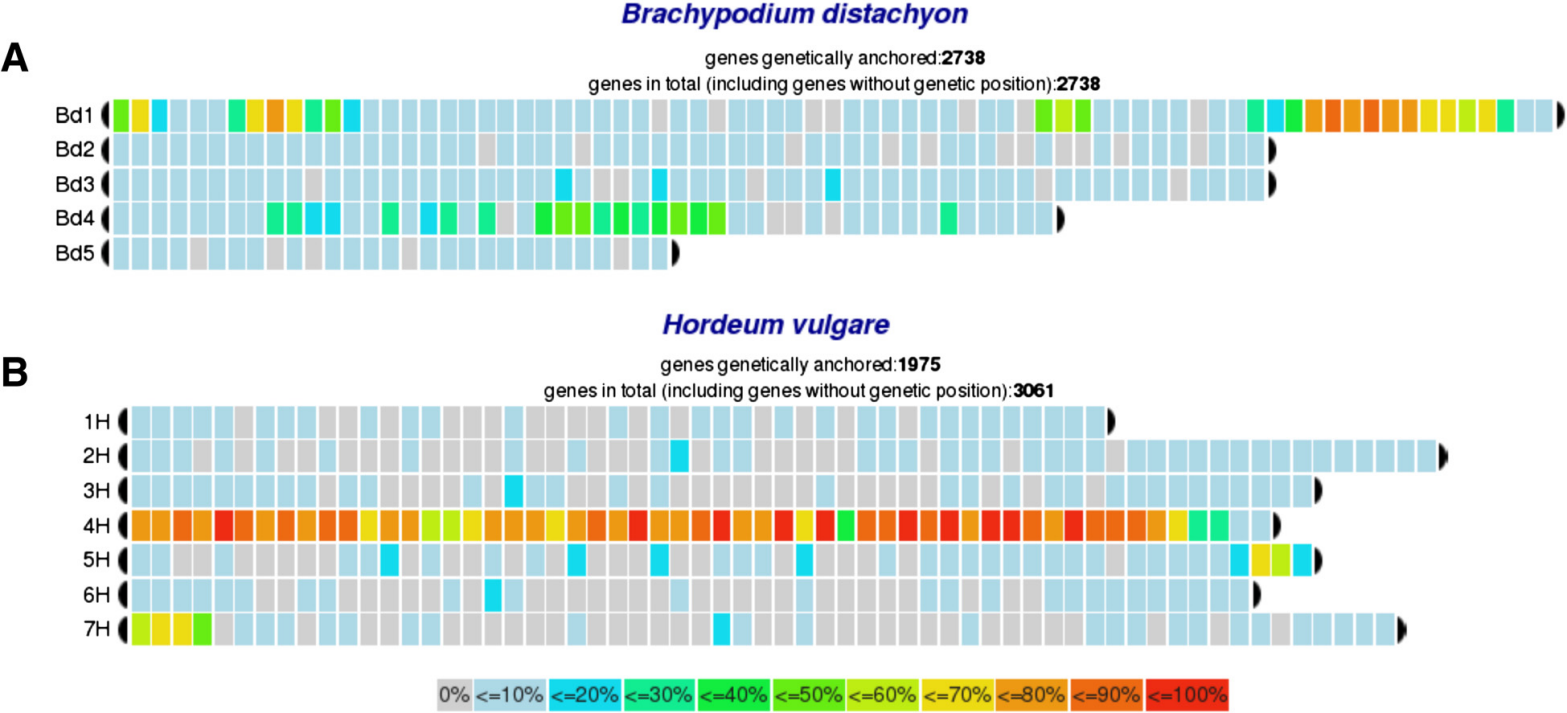
**Synteny between bread wheat chromosome 4A,** ***Brachypodium*** **and barley.** Using *chromoWIZ*, genes from the bread wheat chromosome 4A were mapped against *Brachypodium* **(A)** and against barley **(B)** in order to highlight syntenic regions.

*chromoWIZ* has been repeatedly used to define and refine syntenic regions among related reference genomes [29,30]. For illustration, we used gene models of bread wheat chromosome 4A [6] and to initiated a sequence homology search against *Brachypodium* and barley genes. In total 4,830 genes are annotated on chromosome 4A and the corresponding sequences were extracted and aligned against both genomes using BLASTN (sequence identity of at least 70% and an e-value of 10E-5, best bidirectional hit). We found matches against chromosomes 1 and 4 in *Brachypodium* and a rearrangement of an approximately 3 Mb genomic region that was shifted from the short arm of chromosome 1 to the long arm (Figure 3A). Additionally, in chromosome 4, the centromeric and peri-centromeric near regions were tagged. When bread wheat chromosome 4A was compared against barley, besides the largely homeologous chromosome 4H, syntenic regions on chromosome 5H and chromosome 7H were found, comprising genomic regions of 40 Mb respectively (Figure 3B). These findings are consistent with the documented chromosome rearrangements of bread wheat chromosome 4A [31].

### Use case 4: providing cluster information for tagging genes

**Figure 4:**
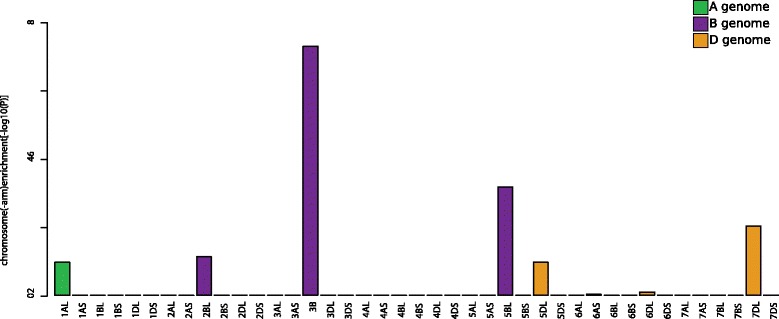
**Chromosome (−arm) enrichment of genes responsive to a fungal pathogen.** Bread wheat chromosome (−arm) enrichment for genes, which were responsive to *Fusarium graminearum*. Chromosome (−arms) 3B, 5BL, and 7DL are found to be significantly enriched for these genes.

Clustering genome-wide expression data into meaningful subsets has become a standard procedure in many transcriptome-oriented studies. Several methods enable to perform such a partitioning of data, e.g. by hierarchical clustering, k-means clustering or network-based approaches. *chromoWIZ* provides support for group-wise analyses as it allows uploading gene-to-group information. The example data for this use case originates from a co-expression network study assessing the effect of fungal pathogens on different bread wheat lines [32]. The five bread wheat lines in this study were characterized by the presence or absence of particular quantitative trait loci (QTL), which confer different resistance levels. This data has been used to infer a co-expression network with the Weighted Correlation Network Analysis approach (WGCNA, [33]). WGCNA can be utilized to find clusters of highly connected genes, so called network modules, based on inferring a correlation-based weighted gene network. After mapping the bread wheat transcriptome data to a *454* sequencing based whole genome assembly [34] and after quantifying the expression using Cufflinks [26], we observed eight different modules which represented distinct expression patterns containing 3,273 genes in total. One module was of particular interest as the related gene expression depicted a pronounced response to the fungal pathogen. The corresponding nucleotide sequences are given in Additional file 3. Using *chromoWIZ* those transcripts were mapped against the bread wheat genome survey sequence [6] by requiring a best bidirectional match and sequence identity of at least 95%. A significant enrichment for chromosome (−arms) 3B, 5BL, and 7DL was found (Figure 4). This is in support of the experimental set-up as one of the major *Fusarium* head blight resistance QTLs (*Fhb1*) that segregates between resistant and susceptible lines and is located on the short arm of chromosome 3B [35].

### Use case 5: *comparative genomics in chromoWIZ for analyzing UDP-gylcosyltransferases*

*chromoWIZ* can be used to detect homologous genes and their locations in the four cereal and model genomes using the implemented BLAST searches. To illustrate this, we searched for *Brachypodium* UDP-glycosyltransferases (UGT) homologous genes in rice, barley, and bread wheat. The *Brachypodium* UGT gene family contains five members of which several encode for the ability to inactivate the mycotoxin deoxynivalenol (Additional file 4) [36]. Deoxynivalenol is a potent inhibitor of protein biosynthesis produced by *Fusarium graminearum*, which is a pathogen also to wheat and barley [37]. The presence/activity of such UGTs may confirm high resistance. Yet, their identification remains challenging also due to the sheer size of the UGT superfamily, which comprises 178 members in *Brachypodium* and probably several hundred in bread wheat [36]. *chromoWIZ* mapped these six genes to the third and fourth bin on chromosome 5 in *Brachypodium*. In order to find putative orthologous genes, we extracted the sequences and mapped them against rice, bread wheat and barley. In barley, matches were found to the 2H (3) and 5H (1) chromosomes using 70% identity and e-value of 10E-5 as search criterions. In addition a match to a yet genetically unanchored gene was found. In rice, matches on chromosome 4 (8) and chromosome 9 (1) were observed, confirming previous findings [36]. In bread wheat matches to 2A (1), 2B (1), 2D (1) and 5A (1) indicate possible homoeologous gene-clusters on linkage group 2, however most genes (13) did not receive any genetic position yet. No matches were observed for chromosome 3B containing the *Fhb1* locus [35], which was previously shown to govern the higher ability to inactivate the toxin [38].

## Discussion

*chromoWIZ* allows searching for candidate genes and visualizing their density and localizations along chromosomes of selected grass genomes. Genes can be searched by using several options, e.g. by gene identifiers, by functional annotation, by sequence homology search or by gene-to-group mappings. The tool is implemented in a flexible way to ensure that novel genomes or updates of existing genomes can be easily undertaken. Export features are provided and extended functionality is activated if gene expression data or clustering information is provided.

### chromoWIZ enables the integration of expression-based information to filter for candidate genes

While there are several tools that provide information, mapping, and visualizations capabilities with respect to syntenic relationships in plant genomes [39,40], there is a lack for tools to query and interactively inspect genetically and physically anchored genes. One of the major advantages of *chromoWIZ* over other tools such as barleymap (http://floresta.eead.csic.es/barleymap/) or IPK Viroblast (http://webblast.ipk-gatersleben.de/barley/) is that expression data can be included to filter by several criteria and thereby selecting the most relevant genes. In addition, clustering information and gene-to-group mappings such as sets of co-expressed genes, selected gene families and/or differentially expressed genes can be included and independently analyzed. The different datasets can be imported by using the Data Manager as intrinsic part of the *chromoWIZ* web application. After uploading the data additional filtering and search options appear on the entry page (Table 1 and Figure 2).

### chromoWIZ enables transferring previous results to the current reference sequences

*chromoWIZ* allows linking gene anchoring information with the annotated gene information and provides access to the gene candidates and their localization as well as to their neighboring genes. With actively ongoing projects and the consequential updates of the reference sequences of bread wheat and barley, data need to be mapped to a common reference sequence to compare previous results against current ones. We demonstrated this approach by using a particular gene co-expression module that comprised the major response of bread wheat genes against a fungal pathogen [32]. As shown in use case 4 *chromoWIZ* allowed transferring previous analysis [32] onto updated resources by mapping from an earlier bread wheat genome draft [34] to more recent chromosome-arm sorted shotgun contigs [6].

### chromoWIZ enables to detect larger syntenic blocks within yet unfinished genomes

For (novel) grass genomes, *chromoWIZ* can be used to detect and analyze syntenic regions with respect to *Brachypodium*, rice, barley, and bread wheat. In use case 3, annotated gene models of bread wheat chromosome 4A were used to detect syntenic regions in comparison to barley and *Brachypodium* (Figure 3). This chromosome is of particular interest, because in most cases barley and wheat chromosomes are collinear [4]. For this specific chromosome syntenic regions appeared also on barley chromosomes 5H and 7H [31]. Furthermore, when arm sorted chromosome datasets become available for a newly sequenced but not yet assembled genome, *chromoWIZ* can help to allocate genes to corresponding syntenic regions in barley, rice, bread wheat, and *Brachypodium*. Thereby, it offers a first glance at the genome structure of these plants, particularly for revealing rearrangements and introgression and to analyze more complex nested syntenic structures.

## Conclusions

*chromoWIZ* provides a valuable and user-friendly interface to access anchored genes for agriculturally important crops and model genomes. By using the different query options it is possible to flexibly narrow down regions of interest and/or gene candidates. With future updates it is planned to include more species and to extend the range of features prior to allow interactive and integrative searches on evolving large and complex crop plant genomes.

## Availability and requirements

*chromoWIZ* is freely available without any restrictions at http://mips.helmholtz-muenchen.de/plant/chromoWIZ/index.jsp.

License: Not required.

Any restrictions to use by non-academics: None.

## Availability of supporting data

The data sets supporting the results of this article are included within the article (and its additional files).

## Additional files

Additional file 1:
**List of genes for use case 1.** List of 19 genes as taken from a particular genomic bin in *Brachypodium* and used for demonstrating the basic functionality of *chromoWIZ*. Additional file 2:
**Barley genes responsive to low potassium for use case 2.** List of barley genes matching transcripts from a study about Tibetan wild barley genotypes under low potassium [28] those were used for integration into the Data Manager. Additional file 3:
**List of fungal pathogen-responsive genes for use case 4.** List of genes that were clustered together in a *Fusarium graminearum* responsive network module as reported in [32]. Additional file 4:
**List of UDP-glycosyltransferases homologs as reported in use case 5.** *Brachypodium* genes of the UDP-glycosyltransferases family and their homologous matches to rice, barley, and bread wheat.

## Abbreviations

BBH: Best bidirectional hit
GO: Gene Ontology
IBSC: International Barley Genome Sequencing Consortium
IWGSC: International Wheat Genome Sequencing Consortium
POPSEQ: Anchoring and ordering NGS contig assemblies by population sequencing
QTL: Quantitative trait loci
UGT: UDP-dependent glycosyltransferases
WGCNA: Weighted Correlation Network Analysis

## References

[1] Arabidopsis Genome Initiative (2000). Analysis of the genome sequence of the flowering plant
Arabidopsis thaliana. Nature.

[2] Michael TP, Jackson S: **The first 50 plant genomes.** *Plant Genome* 2013, **6**(2). https://www.crops.org/publications/tpg/articles/6/2/plantgenome2013.03.0001in.

[3] Morrell PL, Buckler ES, Ross-Ibarra J (2011). Crop genomics: advances and
applications. Nat Rev Genet.

[4] Mayer KF, Martis M, Hedley PE, Simkova H, Liu H, Morris JA, Steuernagel B, Taudien S, Roessner S, Gundlach H, Kubalakova M, Suchankova P, Murat F, Felder M, Nussbaumer T, Graner A, Salse J, Endo T, Sakai H, Tanaka T, Itoh T, Sato K, Platzer M, Matsumoto T, Scholz U, Dolezel J, Waugh R, Stein N (2011). Unlocking the barley genome by chromosomal and
comparative genomics. Plant Cell.

[5] Bolot S, Abrouk M, Masood-Quraishi U, Stein N, Messing J, Feuillet C, Salse J (2009). The ‘inner circle’ of the cereal
genomes. Curr Opin Plant Biol.

[6] International Wheat Genome Sequencing
Consortium (2014). A chromosome-based draft sequence of the hexaploid
bread wheat (Triticum aestivum) genome. Science.

[7] Choulet F, Alberti A, Theil S, Glover N, Barbe V, Daron J, Pingault L, Sourdille P, Couloux A, Paux E, Leroy P, Mangenot S, Guilhot N, Le Gouis J, Balfourier F, Alaux M, Jamilloux V, Poulain J, Durand C, Bellec A, Gaspin C, Safar J, Dolezel J, Rogers J, Vandepoele K, Aury JM, Mayer K, Berges H, Quesneville H, Wincker P (2014). Structural and functional partitioning of bread wheat
chromosome 3B. Science.

[8] Breen J, Wicker T, Shatalina M, Frenkel Z, Bertin I, Philippe R, Spielmeyer W, Simkova H, Safar J, Cattonaro F, Scalabrin S, Magni F, Vautrin S, Berges H, International Wheat Genome Sequencing C, Paux E, Fahima T, Dolezel J, Korol A, Feuillet C, Keller B (2013). A physical map of the short arm of wheat chromosome
1A. PLoS One.

[9] Lucas SJ, Akpinar BA, Kantar M, Weinstein Z, Aydinoglu F, Safar J, Simkova H, Frenkel Z, Korol A, Magni F, Cattonaro F, Vautrin S, Bellec A, Berges H, Dolezel J, Budak H (2013). Physical mapping integrated with syntenic analysis to
characterize the gene space of the long arm of wheat chromosome
1A. PLoS One.

[10] Raats D, Frenkel Z, Krugman T, Dodek I, Sela H, Simkova H, Magni F, Cattonaro F, Vautrin S, Berges H, Wicker T, Keller B, Leroy P, Philippe R, Paux E, Dolezel J, Feuillet C, Korol A, Fahima T (2013). The physical map of wheat chromosome 1BS provides
insights into its gene space organization and evolution. Genome Biol.

[11] Paux E, Sourdille P, Salse J, Saintenac C, Choulet F, Leroy P, Korol A, Michalak M, Kianian S, Spielmeyer W, Lagudah E, Somers D, Kilian A, Alaux M, Vautrin S, Berges H, Eversole K, Appels R, Safar J, Simkova H, Dolezel J, Bernard M, Feuillet C (2008). A physical map of the 1-gigabase bread wheat
chromosome 3B. Science.

[12] Poursarebani N, Nussbaumer T, Simkova H, Safar J, Witsenboer H, van Oeveren J, Dolezel J, Mayer KF, Stein N, Schnurbusch T (2014). Whole-genome profiling and shotgun sequencing delivers
an anchored, gene-decorated, physical map assembly of bread wheat chromosome
6A. Plant Journal.

[13] Ariyadasa R, Mascher M, Nussbaumer T, Schulte D, Frenkel Z, Poursarebani N, Zhou R, Steuernagel B, Gundlach H, Taudien S, Felder M, Platzer M, Himmelbach A, Schmutzer T, Hedley PE, Muehlbauer GJ, Scholz U, Korol A, Mayer KF, Waugh R, Langridge P, Graner A, Stein N (2014). A sequence-ready physical map of barley anchored
genetically by two million single-nucleotide polymorphisms. Plant Physiol.

[14] Mayer KF, Waugh R, Brown JW, Schulman A, Langridge P, Platzer M, Fincher GB, Muehlbauer GJ, Sato K, Close TJ, Wise RP, Stein N, International Barley Genome Sequencing
Consortium (2012). A physical, genetic and functional sequence assembly
of the barley genome. Nature.

[15] Mascher M, Muehlbauer GJ, Rokhsar DS, Chapman J, Schmutz J, Barry K, Munoz-Amatriain M, Close TJ, Wise RP, Schulman AH, Himmelbach A, Mayer KF, Scholz U, Poland JA, Stein N, Waugh R (2013). Anchoring and ordering NGS contig assemblies by
population sequencing (POPSEQ). Plant J.

[16] International Brachypodium
Initiative (2010). Genome sequencing and analysis of the model grass
Brachypodium distachyon. Nature.

[17] Spannagl M, Martis MM, Pfeifer M, Nussbaumer T, Mayer K (2013). Analysing complex Triticeae genomes—concepts and
strategies. Plant Methods.

[18] Martis MM, Zhou R, Haseneyer G, Schmutzer T, Vrana J, Kubalakova M, Konig S, Kugler KG, Scholz U, Hackauf B, Korzun V, Schon CC, Dolezel J, Bauer E, Mayer KF, Stein N (2013). Reticulate evolution of the rye genome. Plant Cell.

[19] Jia J, Zhao S, Kong X, Li Y, Zhao G, He W, Appels R, Pfeifer M, Tao Y, Zhang X, Jing R, Zhang C, Ma Y, Gao L, Gao C, Spannagl M, Mayer KF, Li D, Pan S, Zheng F, Hu Q, Xia X, Li J, Liang Q, Chen J, Wicker T, Gou C, Kuang H, He G, Luo Y (2013). Aegilops tauschii draft genome sequence reveals a gene
repertoire for wheat adaptation. Nature.

[20] Poursarebani N, Ariyadasa R, Zhou R, Schulte D, Steuernagel B, Martis MM, Graner A, Schweizer P, Scholz U, Mayer K, Stein N (2013). Conserved synteny-based anchoring of the barley genome
physical map. Funct Integr Genomics.

[21] International Rice Genome Sequencing
Project (2005). The map-based sequence of the rice
genome. Nature.

[22] Paterson AH, Bowers JE, Bruggmann R, Dubchak I, Grimwood J, Gundlach H, Haberer G, Hellsten U, Mitros T, Poliakov A, Schmutz J, Spannagl M, Tang H, Wang X, Wicker T, Bharti AK, Chapman J, Feltus FA, Gowik U, Grigoriev IV, Lyons E, Maher CA, Martis M, Narechania A, Otillar RP, Penning BW, Salamov AA, Wang Y, Zhang L, Carpita NC (2009). The Sorghum bicolor genome and the diversification of
grasses. Nature.

[23] Nussbaumer T, Martis MM, Roessner SK, Pfeifer M, Bader KC, Sharma S, Gundlach H, Spannagl M (2013). MIPS PlantsDB: a database framework for comparative
plant genome research. Nucleic Acids Res.

[24] Ashburner M, Ball CA, Blake JA, Botstein D, Butler H, Cherry JM, Davis AP, Dolinski K, Dwight SS, Eppig JT, Harris MA, Hill DP, Issel-Tarver L, Kasarskis A, Lewis S, Matese JC, Richardson JE, Ringwald M, Rubin GM, Sherlock G (2000). Gene ontology: tool for the unification of
biology. Gene Ontol Consortium Nat Genet.

[25] Finn RD, Bateman A, Clements J, Coggill P, Eberhardt RY, Eddy SR, Heger A, Hetherington K, Holm L, Mistry J, Sonnhammer EL, Tate J, Punta M (2014). Pfam: the protein families database. Nucleic Acids Res.

[26] Trapnell C, Roberts A, Goff L, Pertea G, Kim D, Kelley DR, Pimentel H, Salzberg SL, Rinn JL, Pachter L (2012). Differential gene and transcript expression analysis
of RNA-seq experiments with TopHat and Cufflinks. Nat Protoc.

[27] Anders S, Pyl PT, Huber W: **HTSeq-a Python framework to work with high-throughput sequencing data.** *Bioinformatics* 2014. http://www.biorxiv.org/content/biorxiv/early/2014/02/20/002824.full.pdf. 10.1093/bioinformatics/btu638 PMC4287950 25260700

[28] Zeng J, He X, Wu D, Zhu B, Cai S, Nadira UA, Jabeen Z, Zhang G (2014). Comparative transcriptome profiling of two Tibetan
wild barley genotypes in responses to low potassium. PLoS One.

[29] Kopecky D, Martis M, Cihalikova J, Hribova E, Vrana J, Bartos J, Kopecka J, Cattonaro F, Stoces S, Novak P, Neumann P, Macas J, Simkova H, Studer B, Asp T, Baird JH, Navratil P, Karafiatova M, Kubalakova M, Safar J, Mayer K, Dolezel J (2013). Flow sorting and sequencing meadow fescue chromosome
4F. Plant Physiol.

[30] Pfeifer M, Martis M, Asp T, Mayer KF, Lubberstedt T, Byrne S, Frei U, Studer B (2013). The perennial ryegrass GenomeZipper: targeted use of
genome resources for comparative grass genomics. Plant Physiol.

[31] Hernandez P, Martis M, Dorado G, Pfeifer M, Galvez S, Schaaf S, Jouve N, Simkova H, Valarik M, Dolezel J, Mayer KF (2012). Next-generation sequencing and syntenic integration of
flow-sorted arms of wheat chromosome 4A exposes the chromosome structure and
gene content. Plant J.

[32] Kugler KG, Siegwart G, Nussbaumer T, Ametz C, Spannagl M, Steiner B, Lemmens M, Mayer KF, Buerstmayr H, Schweiger W (2013). Quantitative trait loci-dependent analysis of a gene
co-expression network associated with Fusarium head blight resistance in bread
wheat (Triticum aestivum L.). BMC Genomics.

[33] Langfelder P, Horvath S (2008). WGCNA: an R package for weighted correlation network
analysis. BMC Bioinformatics.

[34] Brenchley R, Spannagl M, Pfeifer M, Barker GL, D'Amore R, Allen AM, McKenzie N, Kramer M, Kerhornou A, Bolser D, Kay S, Waite D, Trick M, Bancroft I, Gu Y, Huo N, Luo MC, Sehgal S, Gill B, Kianian S, Anderson O, Kersey P, Dvorak J, McCombie WR, Hall A, Mayer KF, Edwards KJ, Bevan MW, Hall N (2012). Analysis of the bread wheat genome using whole-genome
shotgun sequencing. Nature.

[35] Cuthbert PA, Somers DJ, Thomas J, Cloutier S, Brule-Babel A (2006). Fine mapping Fhb1, a major gene controlling fusarium
head blight resistance in bread wheat (Triticum aestivum L.). TAG Theor Appl Genet.

[36] Schweiger W, Pasquet JC, Nussbaumer T, Paris MP, Wiesenberger G, Macadre C, Ametz C, Berthiller F, Lemmens M, Saindrenan P, Mewes HW, Mayer KF, Dufresne M, Adam G (2013). Functional characterization of two clusters of
Brachypodium distachyon UDP-glycosyltransferases encoding putative
deoxynivalenol detoxification genes. Mol Plant Microbe Interact.

[37] Desjardins AE (2006). Fusarium Mycotoxins: Chemistry, Genetics and Biology.

[38] Lemmens M, Scholz U, Berthiller F, Dall'Asta C, Koutnik A, Schuhmacher R, Adam G, Buerstmayr H, Mesterhazy A, Krska R, Ruckenbauer P (2005). The ability to detoxify the mycotoxin deoxynivalenol
colocalizes with a major quantitative trait locus for Fusarium head blight
resistance in wheat. Mol Plant Microbe Interact.

[39] Revanna KV, Munro D, Gao A, Chiu CC, Pathak A, Dong Q (2012). A web-based multi-genome synteny viewer for customized
data. BMC Bioinformatics.

[40] Soderlund C, Bomhoff M, Nelson WM (2011). SyMAP v3.4: a turnkey synteny system with application
to plant genomes. Nucleic Acids Res.

